# Anti-malarial activity of indole alkaloids isolated from *Aspidosperma olivaceum*

**DOI:** 10.1186/1475-2875-13-142

**Published:** 2014-04-14

**Authors:** Talita PC Chierrito, Anna CC Aguiar, Isabel M de Andrade, Isabela P Ceravolo, Regina AC Gonçalves, Arildo JB de Oliveira, Antoniana U Krettli

**Affiliations:** 1 Centro de Ciências da Saúde, de Farmácia, Programa de Pós-Graduação em Ciências Farmacêuticas, Universidade Estadual de Maringá, Avenida Colombo, 5790, CEP 87.020-900 Maringá, PR, Brazil; 2 Faculdade de Medicina, Programa de Pós-Graduação em Medicina Molecular, Universidade Federal de Minas Gerais, Prof. Alfredo Balena, 190, 30130-100 Belo Horizonte, MG, Brazil; 3 Laboratório de Malária, Centro de Pesquisas René Rachou, FIOCRUZ, Av. Augusto de Lima 1715, 30190-002 Belo Horizonte, MG, Brazil

**Keywords:** *Aspidosperma olivaceum*, Indole alkaloids, Aspidoscarpine, Medicinal plants, Antimalarial, *Plasmodium falciparum*

## Abstract

**Background:**

Several species of *Aspidosperma* (Apocynaceae) are used as treatments for human diseases in the tropics. *Aspidosperma olivaceum*, which is used to treat fevers in some regions of Brazil, contains the monoterpenoid indole alkaloids (MIAs) aspidoscarpine, uleine, apparicine, and N-methyl-tetrahydrolivacine. Using bio-guided fractionation and cytotoxicity testing in a human hepatoma cell line, several plant fractions and compounds purified from the bark and leaves of the plant were characterized for specific therapeutic activity (and selectivity index, SI) *in vitro* against the blood forms of *Plasmodium falciparum*.

**Methods:**

The activity of *A. olivaceum* extracts, fractions, and isolated compounds was evaluated against chloroquine (CQ)-resistant *P. falciparum* blood parasites by *in vitro* testing with radiolabelled [^3^H]-hypoxanthine and a monoclonal anti-histidine-rich protein (HRPII) antibody. The cytotoxicity of these fractions and compounds was evaluated in a human hepatoma cell line using a 3-[4,5-dimethylthiazol-2-yl]-2,5 diphenyl tetrazolium bromide (MTT) assay, and the SI was calculated as the ratio between the toxicity and activity. Two leaf fractions were tested in mice with *Plasmodium berghei*.

**Results:**

All six fractions from the bark and leaf extracts were active *in vitro* at low doses (IC_50_ < 5.0 μg/mL) using the anti-HRPII test, and only two (the neutral and basic bark fractions) were toxic to a human cell line (HepG2). The most promising fractions were the crude leaf extract and its basic residue, which had SIs above 50. Among the four pure compounds evaluated, aspidoscarpine in the bark and leaf extracts showed the highest SI at 56; this compound, therefore, represents a possible anti-malarial drug that requires further study. The acidic leaf fraction administered by gavage to mice with blood-induced malaria was also active.

**Conclusion:**

Using a bio-monitoring approach, it was possible to attribute the anti-*P. falciparum* activity of *A. olivaceum* to aspidoscarpine and, to a lesser extent, *N*-methyl-tetrahydrolivacine; other isolated MIA molecules were active but had lower SIs due to their higher toxicities. These results stood in contrast to previous work in which the anti-malarial activity of other *Aspidosperma* species was attributed to uleine.

## Background

Approximately half of the world’s population is at risk for malaria; according to the World Health Organization (WHO), 219 million cases of the disease and an estimated 660,000 deaths were reported in 2010, mostly due to *Plasmodium falciparum*[1]. The recommended treatment for this life-threatening disease is artemisinin-based combination therapy (ACT), but the limited availability of ACT, decreased *P. falciparum* susceptibility to artemisinin derivatives [2,3], and the chloroquine (CQ)-resistance of *Plasmodium vivax* (including in populations in Brazil) [4-7] indicate the need for new and inexpensive drugs for malaria control.

Plants of the *Aspidosperma* genus (Apocynaceae) are used as remedies to treat fever and several human diseases in Brazil [8-11]. They grow in a large variety of habitats in the Americas, from arid fields to the inundated river margins of the Amazon Basin [11]. The main chemical constituents in these plants are monoterpene indole alkaloids (MIAs), which are believed to be primarily responsible for their pharmacological activities (analgesic, anti-inflammatory, bactericidal, central nervous system depressant) [12,13]. Most importantly, several plant extracts and isolated molecules have been reported to have anti-malarial activity in experimental models [14-17]. Several species are believed to be useful against human malaria in the endemic regions of the Amazon basin, among *Aspidosperma sp* and other plants rich in alkaloids [18-21]. However, there have been no ethnopharmacological studies confirming their activities in human malaria in Brazil. Experimental testing with alkaloid-rich fractions of *Aspidosperma nitidum* has supported the belief that it is an anti-malarial remedy, based on its high levels of *in vitro* selectivity indexes in *P. falciparum* and its *in vivo* activity against malaria parasites in mice [21,22]. The active molecule responsible for the anti-malarial activity of *A. nitidum* has not yet been isolated.

**Figure 1:**
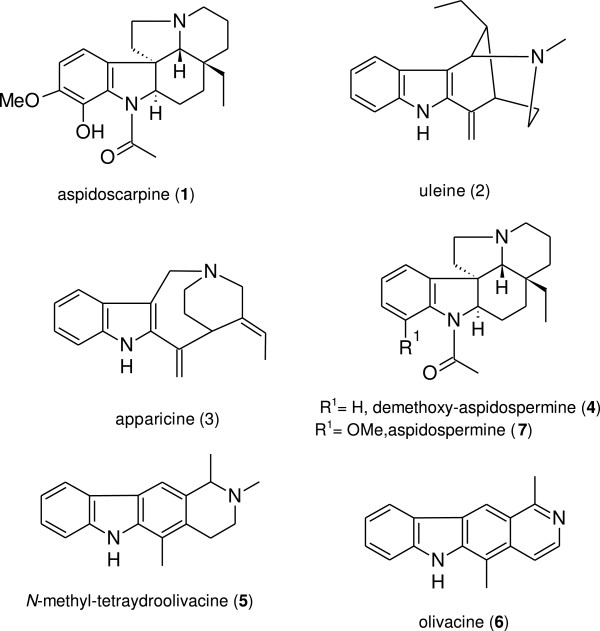
**Anti-malarial monoterpene indole alkaloids detected or isolated from the stem bark and leaves of *Aspidosperma olivaceum.***

Another species used against fever and malaria is *Aspidosperma olivaceum*[23]; the present study examined *A. olivaceum* to determine its most promising anti-malarial compounds by focusing on MIAs that had been previously described in the plant’s bark [8,24]: aspidoscarpine (**1**), uleine (**2**), apparicine (**3**), N-methyl-tetrahydrolivacine (**5**), and olivacine (**6**) (Figure 1). These compounds were further studied using electron-impact mass spectrometry (MS/EI), electron spray ionization (ESI), and nuclear magnetic resonance (NMR). In parallel, cytotoxicity assays were used to analyse the *in vitro* activity of these compounds against *P. falciparum* to determine the compound(s) with the highest therapeutic activity.

## Methods

### General experimental procedures

The isolated compounds were characterized by ^1^H, ^13^C, Distortionless Enhancement by Polarisation Transfer (DEPT), Heteronuclear Multiple Quantum Coherence (HMQC), Heteronuclear Multiple-Bond Correlation (HMBC), and Correlation Spectroscopy (COSY) NMR using a Varian 300 MHz spectrophotometer. The spectra were recorded in CD_3_OD and CDCl_3_ at 300.06 MHz for proton NMR and 75.45 MHz for ^13^C NMR. EI/MS was used for mass spectrometry analysis using a mass spectrometer quadrupole (Thermo Electron Corporation, model DSQ II), with an electron impact energy of 70 eV. The conditions for the analysis were as follows: full-scan mode, positive ions, ion source temperature of 250°C, and mass range of 50–650 Da. The National Institute of Standards and Technology (NIST) Library was used with the direct insertion of the samples in the ion source (Direct Insertion Probe (DIP), Thermo Electron Corporation), with the following temperature program: initial temperature of 70°C, initial time of 20 sec, heating rate of 90°C/min, final temperature of 450°C, and hold time of 180 sec.

The positive mode was used to obtain spectra by electron spray ionisation-mass spectrometry (ESI-MS). The samples were introduced into the spectrometer using a syringe for the “offline” analysis, with probe electron-spray ionization and a triple quadrupole analyser (Micromass®, Quattro MicroTM API). A source temperature of 120°C, desolvation temperature of 350°C, and gas flow of 600 μL/min were used, with adjustments in the capillary voltages and cones specific for each sample. Each spectrum was produced by the accumulation of data for 1 minute [25].

### Plant material

Stem bark and leaves from *A. olivaceum* were collected at the campus of the Universidade Estadual de Maringá, Brazil, in May 2010, where the voucher specimen (HUEM 20500) is deposited; the registry number is 3641 at the IBAMA (Instituto Brasileiro do Meio Ambiente e dos Recursos Naturais Renováveis).

### Extraction and isolation

**Figure 2:**
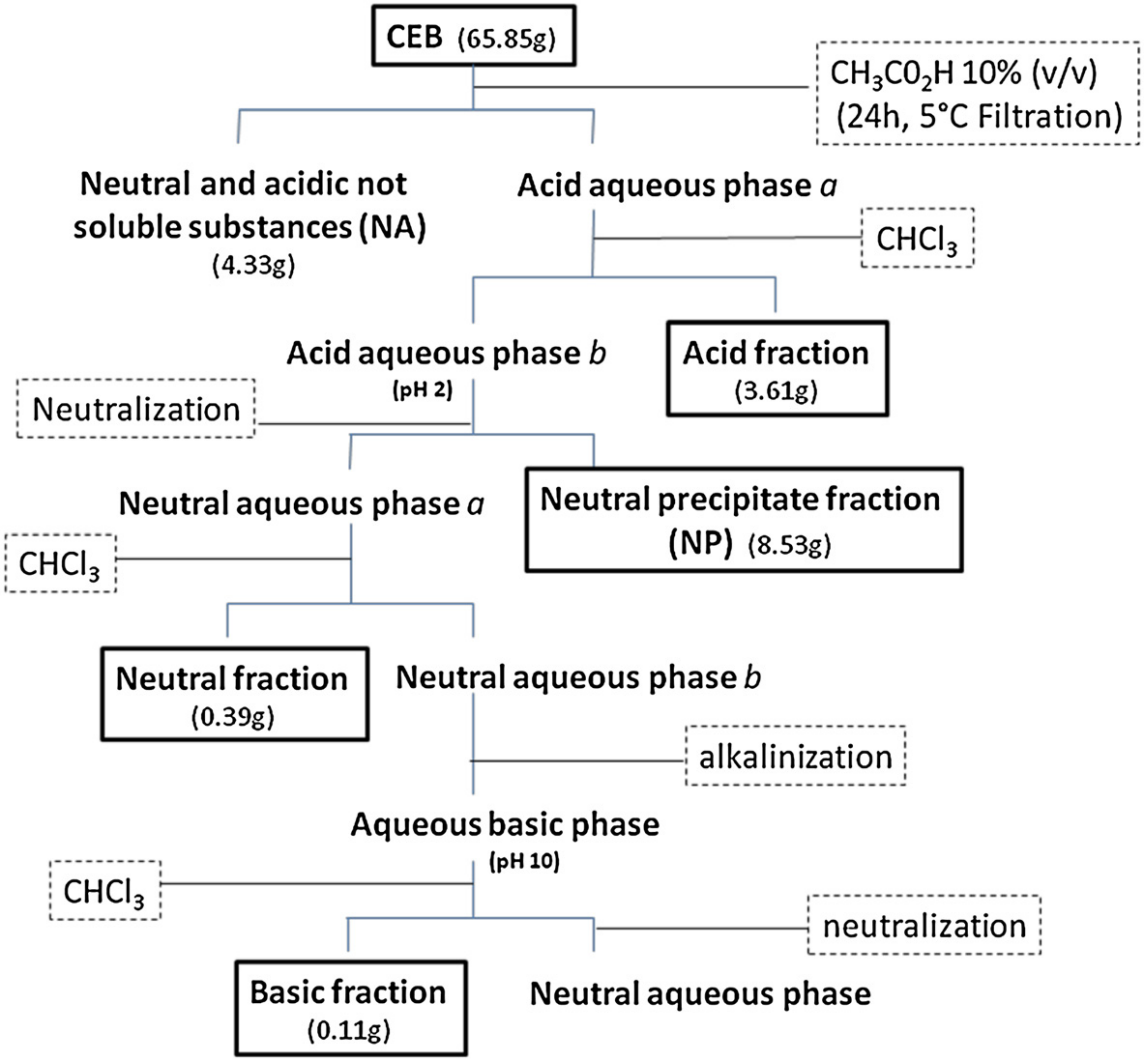
**Acid–base fractionation of the crude extract from the stem bark (CEB) of *Aspidosperma olivaceum.***

The plant materials (635 g of stem bark and 921 g of leaves) were dried in a circulating air oven, ground in a knife mill, and extracted by maceration in methanol for seven days. After the organic solvent was evaporated using a Rotavapor under reduced pressure at 40°C, the crude extracts were lyophilized and subjected to acid–base fractionation [26]. After complete acid–base partition, the stem bark (65.85 g) provided four alkaloid-rich fractions: acidic, neutral precipitate (NP), neutral, and basic. The fractions were lyophilized for further testing; the acidic fraction was also used to further isolate separate compounds as summarized in Figure 2. Additionally, the crude leaf extract (65.96 g) was subjected to a simplified acid–base partition using the same solvents at the appropriate pHs [13,26]; three alkaloid-rich fractions were obtained (acidic fraction, basic residue, and basic fraction) and lyophilized for further testing. The acidic and basic fractions were also used to isolate compounds on preparative thin-layer chromatography (PTLC) plates (20 × 20 cm, with 1.0 mm thickness) of silica gel 60 F254 (Alugram®, Macherey-Nagel, Duren, Germany) activated for 2 hours at 100°C, and each spot was examined under UV light. In addition, the chromogenic reagent *p*-anisaldehyde was used as a developing agent to separate the compounds on TLC with silica gel 60 F254 plates (0.25 mm, Alugram), followed by heating at 105°C. The Dragendorff reagent was used for the initial detection of the compounds.

Compound **(1)** was isolated from the acidic leaf fraction using a mobile phase of chloroform, dichloromethane, and ethyl acetate (4.0:1.0:4.5 v/v, respectively) in an ammonia-saturated environment; compound **(5)** was isolated from the basic fraction using the same mobile phase with the addition of methanol (4.5:4.0:1.0:0.5 v/v, respectively). Compounds **(1)**, **(2)**, and **(3)** were isolated from the stem bark, and the mobile consisted of chloroform, toluene, and methanol (10:2.0:0.5 v/v, respectively) in an ammonia-saturated environment. All of the compounds were dissolved in CHCl_3_:MeOH (1:1 v/v) and filtered through a sintered funnel, and the organic solvent was evaporated under reduced pressure. The samples were then subjected to NMR and MS analyses and lyophilized for biological testing.

### *In vitro* testing against *Plasmodium falciparum* blood stages

The activities of the *A. olivaceum* extracts, fractions, and isolated compounds were evaluated against CQ-resistant *P. falciparum* (clone W2) blood parasites, which were cultured as previously described [27], with modifications [28]. Immediately after synchronization in sorbitol [29], the concentrated ring stages were incubated with the test compounds, which had been previously solubilised in 0.05% dimethylsulfoxide (DMSO) (v/v) using six serial concentrations (0.75-50 μg/mL). Each test was performed in triplicate; CQ was tested in parallel as an anti-malarial control. The effects of the compounds against *P. falciparum* were measured using: (i) the [^3^H]- hypoxanthine incorporation assay [30] and (ii) commercially available monoclonal antibodies specific for histidine-rich protein (HRPII), a histidine- and alanine-rich parasite protein (MPFM ICLLAB-55A® and MPFG55P ICLLAB®, USA), as described previously [31]. The incorporation of [^3^H]-hypoxanthine was measured in a beta cell counter (PerkinElmer, Waltham, MA, USA). The binding of anti-HRPII antibody was quantified at 450 nm using a spectrophotometer (SpectraMax340PC384, Molecular Devices). The half-maximal drug inhibitory response (IC_50_) was estimated by curve fitting using software from OriginLab Corporation (Northampton, MA, USA). The results were compared with drug-free control wells, which were considered to represent 100% parasite viability.

### Cytotoxicity testing *in vitro*

The cytotoxicity testing was performed in two cell lines: human hepatoma cells (HepG2) and normal kidney glomerular cells (BGM). Both of these cell lines were cultured in 75-cm^2^ sterile flasks in Roswell Park Memorial Institute (RPMI) 1640 medium supplemented with 10% heat-inactivated foetal calf serum and 40 mg/L gentamicin in a 5% CO_2_ atmosphere at 37°C. When confluent, the cell monolayer was washed with culture medium, trypsinized, distributed in a flat-bottomed 96-well plate (5 × 10^3^ cells/well), and incubated for 18 h at 37°C for cell adherence [32]. The compounds (20 μL) were diluted to various concentrations (1–1000 μg/mL) and incubated with the cells for 24 h in a 5% CO_2_ atmosphere at 37°C. A 3-(4,5-dimethylthiazol-2-yl)-2,5-diphenyltetrazolium bromide (MTT) solution (5 mg/mL; 20 μL/well) was added to evaluate mitochondrial viability; after a further 3 h incubation, the supernatants were carefully removed, 100 μL of DMSO was added to each well, and the reactions were mixed to solubilize the formazan crystals. The optical density was determined at 570 nm and 630 nm to measure the signal and background, respectively (SpectraMax340PC384, Molecular Devices). The cell viability was expressed as a percentage of the control absorbance in the untreated cells after subtracting the appropriate background. The minimum lethal dose for 50% of the cells (MLD_50_) was determined as described [33], and the values were used to calculate the selectivity index (SI), which is the ratio between the cytotoxicity and the activity.

### Anti-malarial tests against *Plasmodium berghei* in mice

The suppressive test of parasite growth in mice was performed as described [34], with modifications [15]. Briefly, adult Swiss outbred mice (20 ± 2 g weigh) were inoculated by intraperitoneal injection with 1 × 10^5^*P. berghei* (ANKA strain)-infected red blood cells. The mice were maintained together for at least 2 h before, divided randomly into groups of six animals per cage, then treated with 100 or 200 mg/kg of each compound diluted in 3% DMSO in water (v/v), by daily gavage for three consecutive days. Two control groups (5 mice each) were used in parallel; one group was treated with CQ (20 mg/kg), and one group received the drug vehicle alone. Blood smears were prepared daily on post-infection days 5 through 10, methanol-fixed, stained with Giemsa, and examined microscopically in coded smears for parasitaemia counts. The percent inhibition of parasite growth was calculated relative to the untreated control group (considered 100% growth). For the Mann–Whitney test, the GraphPad Prism 5 program was used. Mortality was monitored daily.

The use of laboratory animals was approved by the Ethics Committee for Animal Use of the Oswaldo Cruz Foundation - Fiocruz (CEUA L-0046/08).

## Results and discussion

The extracts of *A. olivaceum* were tested *in vitro* against CQ-resistant *P. falciparum* (W2 clone) blood parasites; most were active with IC_50_ values below 10 μg/mL (Table 1). In the HRPII assay, the bark crude extract and its fractions (acidic and neutral precipitate) showed IC_50_ values below 5.0 μg/mL; the leaf crude extract, the basic residue, and the acid fraction were also active. The purified compounds from the plant bark and leaves had IC_50_ values between 3.2 and 4.4 μg/mL Two plant fractions were also tested in vivo, one was active (Table 2).

**Table 1:** ***In vitro *** **activity of *Aspidosperma olivaceum *** **extracts and fractions against *P. falciparum *** **chloroquine-resistant blood parasites (W2 clone), cytotoxicity (MDL**_**50**_**) to a human hepatoma cell line (HepG2), and selectivity index (SI), the ratio between the MDL**_**50 **_**and IC**_**50**_

Compound*	**MDL**_**50 **_**(μg/mL)**	**IC**_**50 **_(μg/mL)**		SI***
		**[**^**3**^**H]-hypoxanthine**	**Anti-HRPII**	
**Bark extract and fractions**				
Crude bark extract	177 ± 4.0	10.6 ± 3.0	9.9 ± 4.5	17
Acid fraction	330 ± 54.0	6.4 ± 2.3	5.5 ± 2.5	52
Neutral fraction	20 ± 3.0	2.0 ± 0.2	2.1 ± 0.5	10
Basic fraction	27 ± 0.0	4.3 ± 2.5	3.8 ± 0.4	<6
Neutral precipitate (NP)	455 ± 100.0	6.7 ± 3.3	5.0 ± 2.5	68
**Leaf extract and fractions**				
Crude leaf extract	904 ± 135.4	7.2 ± 2.3	9.2 ± 5.4	126
Basic residue	415 ± 98.8	4.5 ± 0.7	2.0 ± 1.5	92
Acid fraction	441 ± 59.0	8.5 ± 3.5	5.3 ± 1.1	52
Pure substance from extracts*				
** (1)** Aspidoscarpine #	301 ± 24.0	5.4 ± 2.5	4.4 ± 0.8	56
** (2)** Uleine	52 ± 10.0	7.0 ± 0.0	3.2 ± 1.8	18
** (3)** Apparacine	41 ± 2.8	3.0 ± 1.4	3.2 ± 2.7	14
** (5** N-methyl-tetrahydrolivacine	126 ± 6.0	5.7 ± 3.3	4.0 ± 2.8	22

*The steps for the bio-fractionation of the extracts are summarized in Figure 1.

**IC_50_ = dose inhibiting 50% of parasite growth, evaluated in three or four different experiments for each test.

***SI = values obtained using the [^3^H]-hypoxanthine test; toxicity considered present at an SI <10.

#Compound (1) was isolated from the leaves and bark. The compounds are numbered 1, 2, 3, and 5 as in Figure 2; their complete names and structures are listed in Figure 1.

**Table 2:** **Activity anti-*P. falciparum *** **(IC**_**50**_) **of molecules isolated from wood and/or barks of *Aspidosperma sp*, data described previously in the literature**

***Aspidosperma sp.***	**Molecule**	**IC**_**50 **_**(μg/mL)**	**Reference**
*A. desmanthum*	Aspidoscarpine	0.007	Andrade-Neto *et al.*[19]
*A. marcgravianum*	Aricin	0.3	Passemar *et al.*[39]
	Tchibangensin	0.13	
	Tetrahydrousambarensin	0.26	
*A. oblongum*	Usambarensin	0.23	Passemar *et al.*[39]
	Ochrolifuanin A	0.47	
*A. parvifolium*	Uleine	0.98 ± 0.20*	Oliveira *et al.*[17]
*A. olivaceum*	Olivacine	0.34	Rocha e Silva *et al.*[35]
*A. ulei*	20-*epi*-dasycarpidone	4.5	Dos Santos Torres *et al.*[40]
*A. vargasii*	Ellipticine	0.2	Rocha e Silva *et al.*[35]
		0.018	Andrade-Neto *et al.*[19]

*IC_50_ in μM.

Most of the extracts displayed low toxicity, especially the crude leaf extract and its fractions, with SIs up to 126 (Table 1). However, the neutral and basic fractions from the plant bark were toxic (SI below 10). Among the purified fractions, compound (**1**), aspidoscarpine, was the most promising (SI of 56), followed by compound (**5**) with a SI of 22; compounds **(2)** and **(3)** showed SI values of 18 and 14, respectively, and were considered moderately toxic.

This is the first report regarding the isolation of two MIAs (**1** and **5**) from the leaves of *A. olivaceum*. The compounds aspidoscarpine, uleine, olivacine, and ellipticine had previously been characterized in other species of *Aspidosperma*. These compounds were also present in *A. olivaceum* and tested against *P. falciparum in vitro* in the present study*,* but some of the compounds were inactive or toxic (Table 2). Novel molecules that are active *in vitro* have been identified in *Aspidosperma parvifolium, A. olivaceum, Aspidosperma vargasii*, and *Aspidosperma ramiflorum*. Extracts from *A. vargasii* and *A. olivaceum* have been tested and found to be active in mice infected with *P. berghei*[35]*.*

The potential of the active acid fraction to induce mutagenic and genotoxic effects *in vitro* was evaluated with Ames tests [36], which were performed at the Genotox-Royal Institute, Rio Grande do Sul, Brazil (contract GT00742). These tests utilized five strains of *Salmonella typhimurium* with different mutations and drug concentrations of up to 2,500 μg/plate in the absence and presence of the metabolizing rat liver fraction. None of the compounds exhibited mutagenic or genotoxic potential.

**Figure 3:**
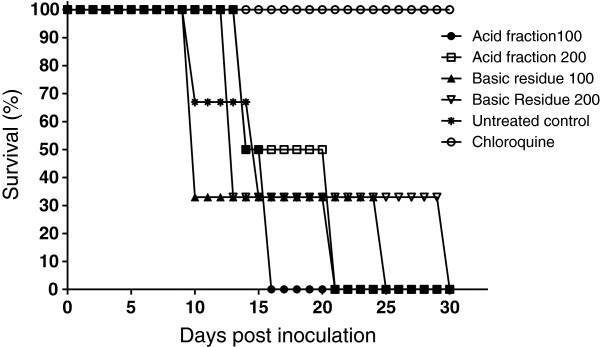
**Survival of mice infected with *Plasmodium berghei *** **(ANKA strain) after oral treatment with *Aspidosperma olivaceum *** **fractions or chloroquine (20 mg/kg) and of non-treated control mice (5 mice per group).**

The acidic fraction and the basic residue from the leaves of *A. olivaceum* were evaluated for activity against *P. berghei* in mice with blood-induced malaria. The acidic fraction was active and reduced parasitaemia by as much as 79% at the 100 mg/kg dose; the 200 mg/kg dose reduced parasitaemia by as much as 58% (Table 2). Only CQ, a control anti-malarial, caused 100% suppression of parasitaemia, and all of the mice survived until 30 days post infection, the last day of observation. Examining the time elapsed to reach 50% survival, half of the control non-treated malaria group survived 10 days of infection, and half of the mice treated with 200 mg/kg of the acid fraction survived approximately 21 days; half of the mice receiving 100 mg/kg survived 16 days (Figure 3). Nevertheless, these differences were not statistically significant, and all of the animals died of malaria, except the animals in the CQ-treated group.

**Figure 4:**
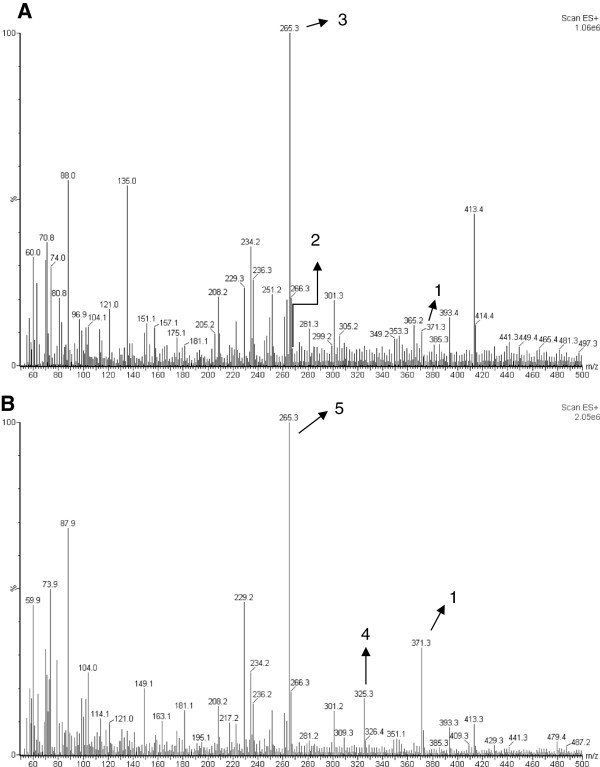
**Off-line ESI-MS of crude extract from the stem bark (A) and leaves (B) of *Aspidosperma olivaceum*.** The ion at *m/z* 371 [M + H]^+^ corresponded to aspidoscarpine (**1**), the ion at *m/z* 267 [M + H]^+^ corresponded to uleine (**2**), the ion at *m/z* 265 [M + H]^+^ corresponded to apparicine (**3**), the ion at *m/z* 326 [M + H]^+^ corresponded to demethoxy-aspidospermine (**4**), and the ion at *m/z* 265 [M + H]^+^ in the crude leaf extract corresponded to *N*-methyl-tetrahydrolivacine (**5**).

The chemical studies of *A. olivaceum* allowed us to isolate and characterize four MIAs using EI-MS, ESI-MS, and NMR: aspidoscarpine (**1**), uleine (**2**), apparicine (**3**), and N-methyl-tetrahydrolivacine (**5**) (Figures 1, 2, 3, 4). These compounds had been previously described in the bark of *A. olivaceum*[8] and in other *Aspidosperma* genera species [15,16,22]. They were isolated from *A. olivaceum* stem bark and leaves and identified by TLC fingerprints and ESI-MS; several of the predominant ion fragments found in the crude extract and fractions were found in the isolated compounds (**1**–**3** and **5**), suggesting that these compounds were present in the crude samples.

A preliminary off-line ESI-MS analysis of the crude extract from *A. olivaceum* stem bark showed mass peaks at *m/z* 371, 267, and 265 [M + H]^+^, which corresponded to the alkaloids aspidoscarpine (**1**), uleine (**2**), and apparicine (**3**) (Figure 4A); the ESI-MS/MS experiments from these isolated compounds showed ion daughters at *m/z* 124, 236, and 134 [M + H]^+^. In the acid fraction of the stem bark, peaks were detected at *m/z* 371, 325, 267, 265, and 247 [M + H]^+^, which corresponded to aspidoscarpine (**1**), demethoxy-aspidospermine (**4**), uleine (**2**), apparicine (**3**), and olivacine (**6**), respectively. In the basic fraction, the ions were *m/z* 265 and 247 [M + H]^+^, which corresponded to apparicine (**3**) and olivacine (**6**), respectively. In the neutral precipitate, we detected the ions *m/z* 371, 265, and 247 [M + H]^+^, which corresponded to aspidoscarpine (**1**), apparicine (**3**), and olivacine (**6**), respectively, whereas ions with *m/z* 265, 247, and 355 [M + H]^+^, which corresponded to apparicine (**3**), olivacine (**6**), and aspidospermine (**7**), respectively, were identified in the neutral fraction. The crude leaf extract contained peaks at *m/z* 371, 325, and 265 [M + H]^+^, which corresponded to aspidoscarpine (**1**), demethoxy-aspidospermine (**4**), and *N*-methyl-tetrahydrolivacine (**5**), respectively (Figure 4B). The ESI-MS/MS experiments on isolated compounds **1** and **5** showed respective ion daughters at *m/z* 124 and 219 [M + H]^+^*.*

**Figure 5:**
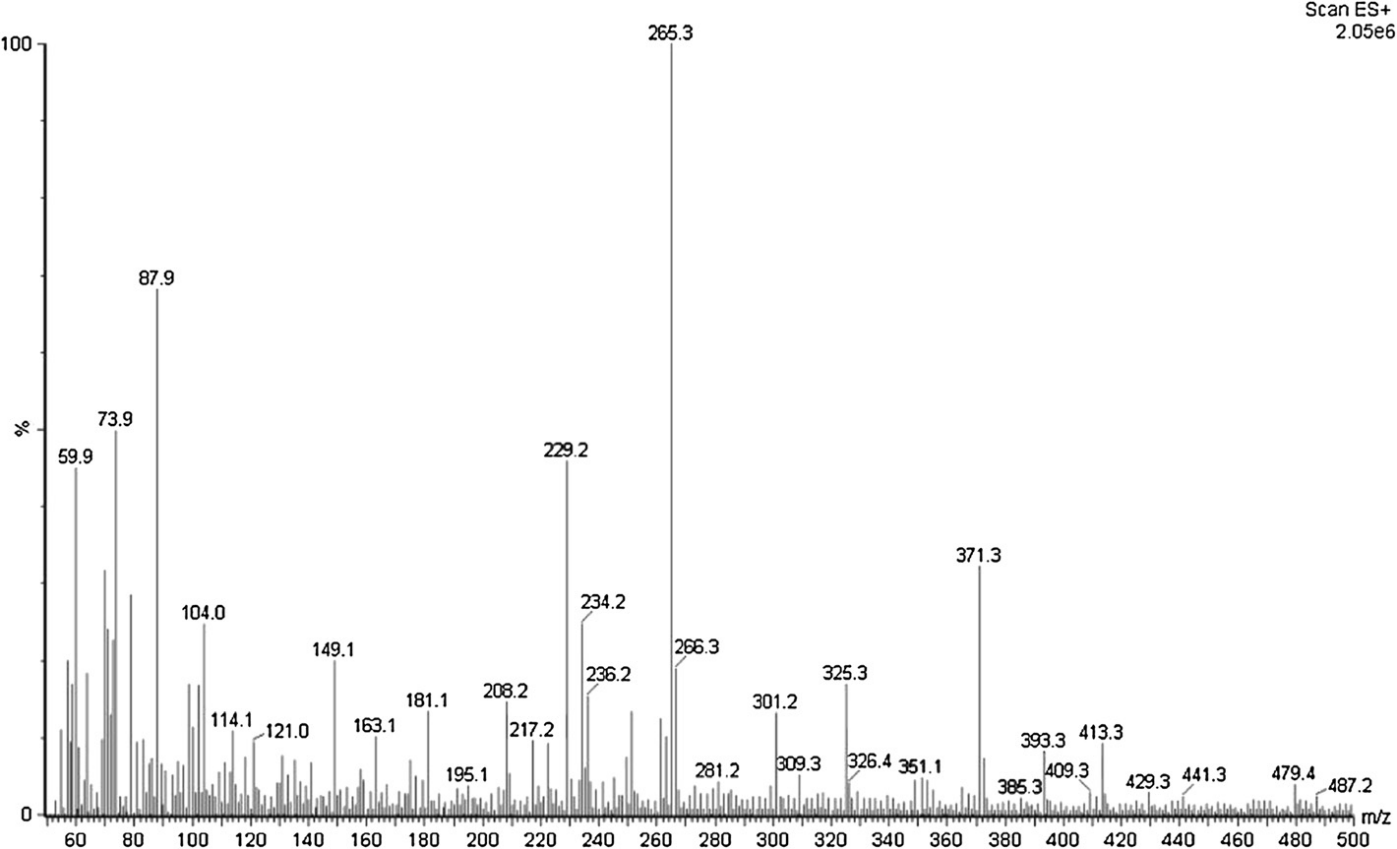
**Off-line ESI-MS of the acid fraction from the leaves of *Aspidosperma olivaceum*.** The ion at *m/z* 371 [M + H]^+^ corresponded to aspidoscarpine (**1**); the ion at *m/z* 326 [M + H]^+^ corresponded to demethoxy-aspidospermine (**4**), and the ion at *m/z* 265 [M + H]^+^ corresponded to *N*-methyl-tetrahydrolivacine (**5**).

The leaf acidic fraction revealed ions at *m/z* 371, 325, and 265 [M + H]^+^, which corresponded to aspidoscarpine (**1**), demethoxy-aspidospermine (**4**), and *N*-methyl-tetrahydrolivacine (**5)**, respectively (Figure 5). The basic residue and basic fraction showed ions at *m/z* 371, 325, and 265 [M + H]^+^, which corresponded to aspidoscarpine (**1**), demethoxy-aspidospermine (**4**), and *N-*methyl-tetrahydrolivacine (**5**), respectively. The results of the off-line ESI-MS analysis of the fractions obtained from the leaf crude extract agreed with the results obtained by the isolation of the compounds described below.

**Figure 6:**
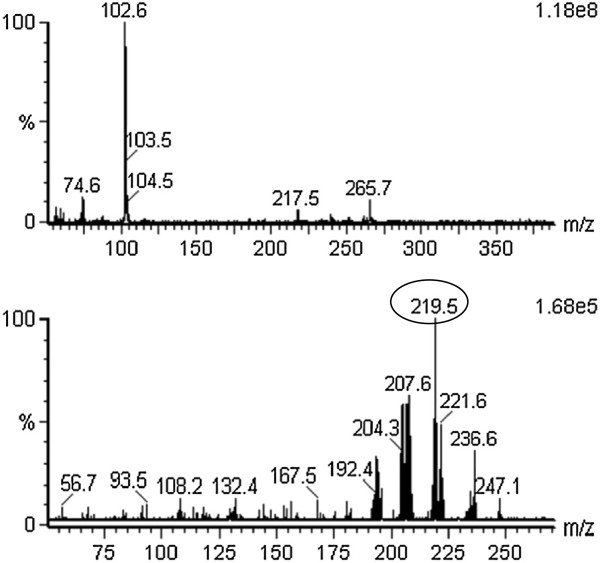
**Off-line ESI-MS of (A) crude extract from the leaves of *Aspidosperma olivaceum *and (B) the daughter ions from fragment *m/z *265 [M + H]**^**+**^**.** The daughter ion at *m/z* 219 [M + H]^+^ was the most abundant species.

**Figure 7:**
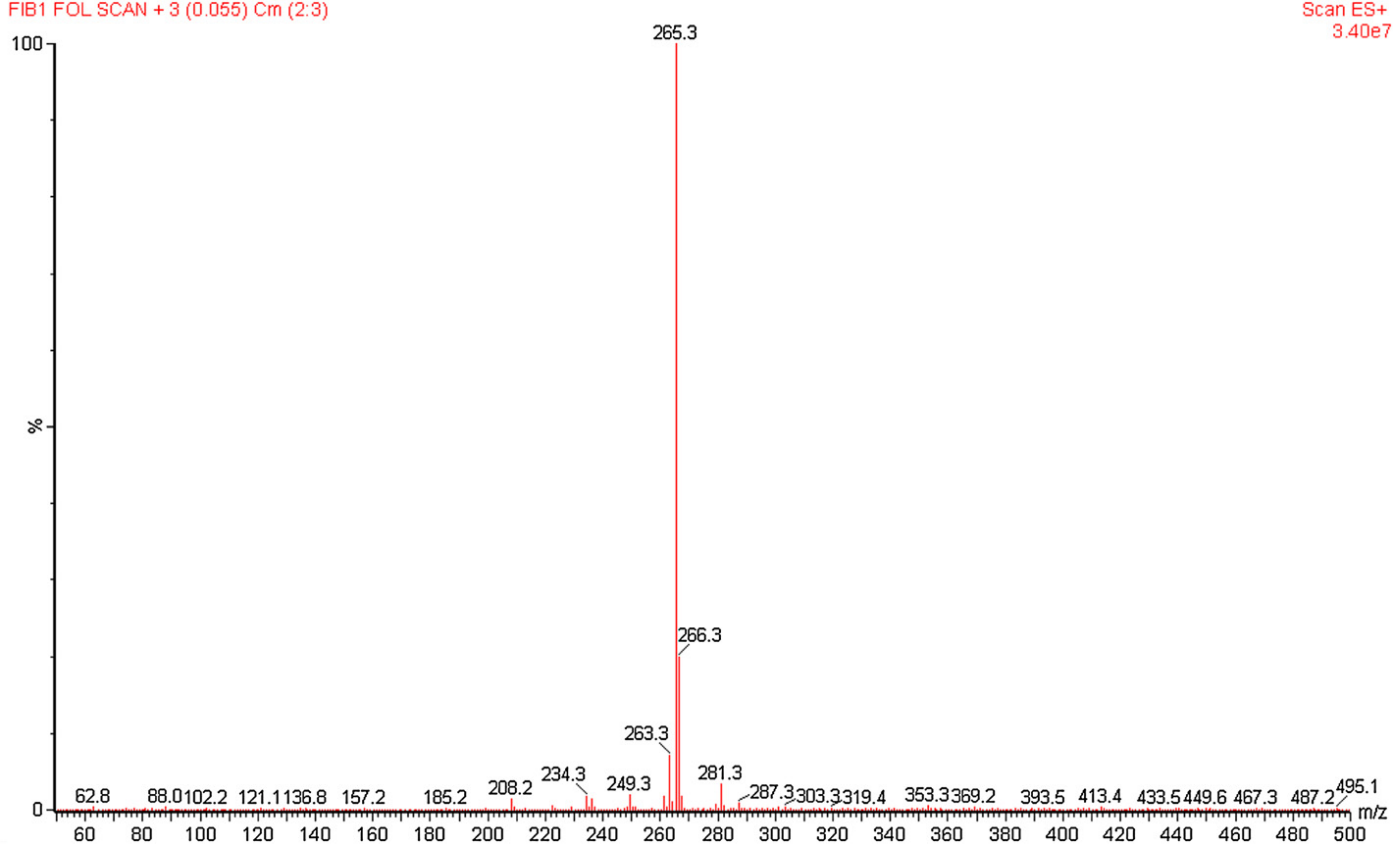
**Off-line ESI-MS of (A) *N*-methyl-tetrahydrolivacine (5) isolated from the leaves of *Aspidosperma olivaceum*.**

**Figure 8:**
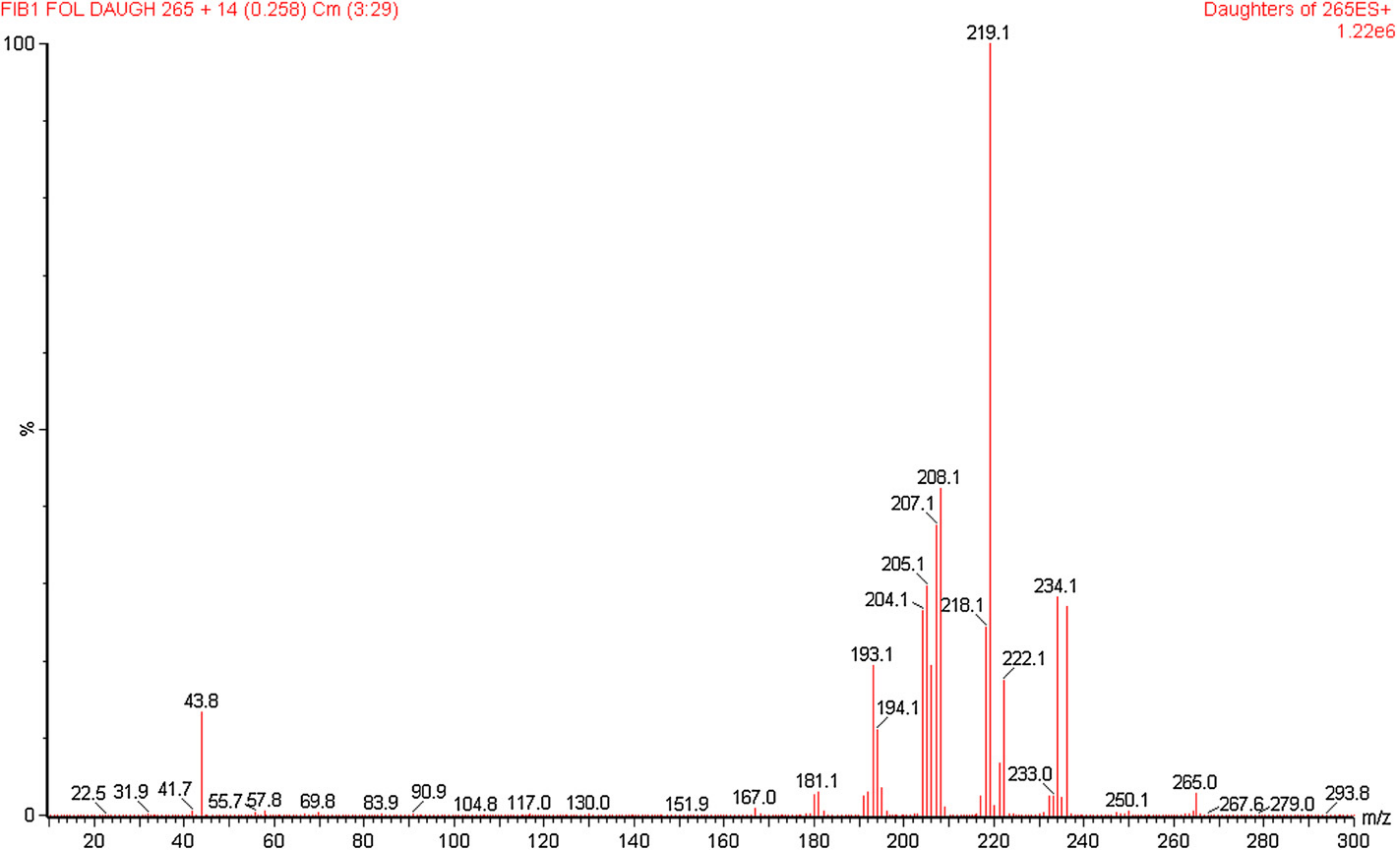
**Off-line ESI-MS/MS of fragment *m/z *265 [M + H]**^**+**^**.** The daughter ion at *m/z* 219 [M + H]^+^ was the most abundant species.

Although apparicine (**3**) and *N*-methyl-tetrahydrolivacine (**5**) have the same mass and the same pseudomolecular ion *m/z* 265 [M + 1]^+^, they could be distinguished by the colours developed on TLC in the presence of a *p-*anisaldehyde chromogenic reagent (compound **3** is a brown spot, while **5** is violet) and by their predominant daughter ions, such as the ions at *m/z* 134 and 219 [M + H]^+^, respectively. The presence of compound **3** in the crude extract and fractions of the stem bark was confirmed by TLC and ESI-MS. Compound **5** was present in the crude leaf extract and all of its fractions, as confirmed by TLC and ESI-MS/MS. Selecting the ion at *m/z* 265 [M + H]^+^ from the off-line mass spectrum of the crude leaf extract, it was possible to observe the characteristic fragmentation similar to the ESI-MS mass spectrum of *N*-methyl-tetrahydrolivacine (**5**) isolated from the alkaloid basic leaf fraction: the main ions at m/z 236, 219 and 208 [M + H]^+^ are shown in Figures 6, 7 and 8.

The identification of the isolated compounds was performed as follows. Compound **1** was identified by EI/MS at 70 eV by the following *m/z* (%): 370 [M + .]; 342 (10), 176 (2), 124 (100); and ESI-MS *m/z* 371 [M + H]^+^ with daughter ion *m/z* 124 [M + H]^+^. The NMR data were consistent with the literature [37] for aspidoscarpine (**1**), which was isolated from both the leaves and stem bark, in agreement with the MS and NMR data and literature [25]. Compound **2** was identified by EI/MS at 70 eV by the following *m/z* (%): 266 [M + .]; 266 (100), 265 (26), 237 (60), 223 (46), 209 (92), 208 (58), 194 (75), 180 (57), 167 (30); and ESI-MS *m/z* 267 [M + H]^+^ with daughter ion *m/z* 236 [M + H]^+^. The NMR data available in the literature confirmed this compound as uleine (**2)**[38]. Compound **3** was identified by EI/MS at 70 eV by the following *m/z* (%): 264 [M + .] (21); 264 (100), 249 (42), 235 (38), 222 (52), 208 (66); and ESI-MS *m/z* 265 [M + H]^+^ with daughter ion *m/z* 134 [M + H]^+^. The NMR data were consistent with the literature [38] for apparicine (**3**). Compound **5** was identified by EI/MS by 70 eV by the following *m/z* (%): 264 [M + .] (2); [M-1] 263 (3); [M-15] 249 (100), 247 (5), 218 (2), 206 (2), 205 (2), 204 (3), 132 (2), 124 (4), 117 (7); and ESI-MS *m/z* 265 [M + H]^+^ with daughter ion *m*/z 219 [M + H]^+^. The NMR data indicated that this compound was *N*-methyl-tetrahydrolivacine (also known as guatambuine), which was consistent with the literature [25].

## Conclusion

Through a bio-monitoring approach and an evaluation of their anti-*P. falciparum* therapeutic activities, several extracts and fractions of *A. olivaceum* were isolated and found to be active; this report was the first successful isolation of MIA compounds from plant leaves. The MIA aspidoscarpine (**1**) was considered to be the most promising based on its high SI against *P. falciparum* and its low cytotoxicity *in vitro*. Other MIAs were isolated and were active *in vitro*, consistent with previous work on plant bark (Table 2). The leaf acidic fraction was also active against malaria in mice with *P. berghei,* reducing parasitaemia and delaying malaria mortality. The present data confirmed the previously published results from Andrade-Neto *et al.*[15] using *A. desmanthum*, which also suggested that aspidoscarpine was the most active molecule *in vitro* against *P. falciparum* (IC_50_ 0.007)*.* Together, these results indicate that aspidoscarpine may be an important anti-malarial lead that should be further analysed.

## Competing interests

The authors declare that they have no competing interests.

## Authors’ contributions

TPCC carried out the extraction, fractionation and isolation of the compounds; ACCA and IPC performed the biological *in vitro* assays and performed the statistical analysis; IMA performed the biological *in vivo* tests; AOB and RACG conceived the chemical study and participated in its design; AUK was the project leader, conceived and was responsible for the study and the pharmacological tests. All of the authors read and approved the final manuscript.
